# Tracking cell fate through morphodynamics

**DOI:** 10.1038/s44320-026-00244-3

**Published:** 2026-08-27

**Authors:** Julia Dorr, Brian J Mitchell

**Affiliations:** Northwestern University, Feinberg School of Medicine, Department of Cell and Developmental Biology, Chicago, IL USA

**Keywords:** Computational Biology, Development

## Abstract

Dorr and Mitchell discuss the study by Tolonen et al where they employ morphodynamic profiling with modeling, which reveals changes in cell and nuclear geometry that encode cell fate during mucociliary epithelial differentiation.

**Figure:**
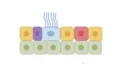

## Teaser

Cell state transitions are an important feature of both development and disease. Most of what we know about these transitions derives from single cell molecular measurements which only capture static snapshots of a highly dynamic process. The field of morphodynamics broadly exemplified by the recent work of Tolonen et al aims to address these shortcomings by using live imaging to capture the morphological changes associated with differentiating cells. Their goal is to create a computational pipeline to analyze phenomics in order to determine cell fate, ultimately increasing understanding of cell state transitions and functioning tissues.

Cells differentiate to serve specialized functions in their environment, which changes how they look, move and act. Even though cell state transitions are central to understanding developing tissues, the underlying morphodynamics are not well characterized, as cell identity and development is currently primarily understood at the molecular level. Single-cell data, such as RNA sequencing has provided in-depth gene expression profiles which reveal snapshots of cell state at a given moment and have provided significant insight into distinct transition states and regulatory networks (Griffiths et al, 2018; Lee et al, 2023). Advances in computational methods have made it possible to connect these snapshots and construct inferred trajectories of cell fate (Trapnell et al, 2014). However, they cannot recapitulate it fully, leaving important information about cell transitions unknown. In Tolonen et al, the authors aim to address this fundamental question in developmental biology and build on previous work using segmentation and tracking to follow cells from the multipotent progenitor state to their terminal identity. To do this, they developed a quantitative and computational pipeline that can derive information from morphometrics obtained from live imaging with the goal of predicting cell fate (Tolonen et al, 2026). This will not only provide important insight into development but will also identify the critical time points of transition that, when coupled with omics-level approaches in the future, should provide more depth to our understanding of these cell fate transitions.

**Figure 1:**
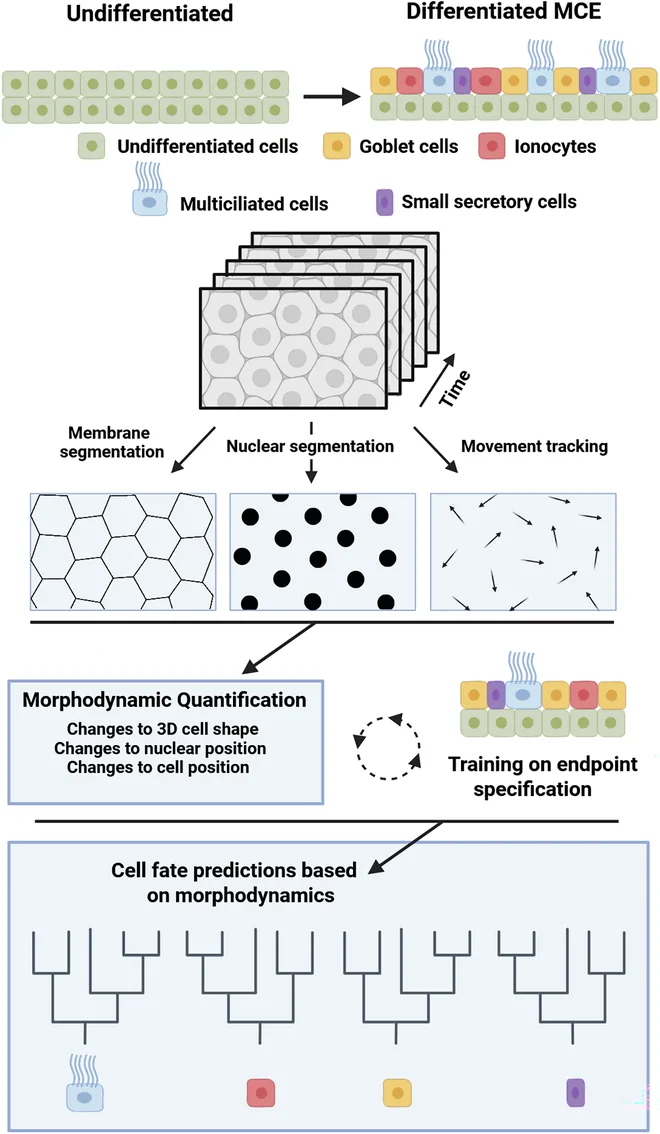
Model for using morphodynamics to determine cell fate. (Top) The system used by Tolonen of *Xenopus* mucociliary epithelium (MCE) that goes from undifferentiated to completely differentiated with numerous cell types in 1 day. (Middle) Depiction of the analysis and training pipeline used to measure morphodynamic changes. (Bottom) Overall output of utilizing morphodynamic changes to predict cell fate trajectories. Created in BioRender. Mitchell, B. (2026) https://BioRender.com/wsshet8.

Tolonen et al take advantage of the *Xenopus* mucociliary epithelium (MCE), which is a rapidly differentiating tissue that results in a multilayered complex epithelium (Fig. 1). A particularly useful feature is that this tissue can be excised as an ectodermal cap and attached to fibronectin coated glass bottom dishes where it will undergo the entire differentiation process (Dingwell and Smith, 2018). This system recapitulates many aspects of MCE development, and the resulting tissue is quite similar to other MCEs, such as the mammalian airway epithelium. Importantly, while this is a multilayered 3D culture, it is relatively thin, allowing for high spatial and temporal resolution during long-term live-imaging. During the developmental window of their analysis (~22 h), the MCE produces a mature bilayered tissue with a variety of specialized cells, including multiciliated cells (MCCs), small secretory cells (SSCs), ionocytes (ICs), goblet cells and basal stem cells, some of which have distinct morphologies and cell movement trajectories that are utilized as important features of cell tracking (Walentek, 2021).

Using embryos that were injected with fluorescent nuclear and membrane markers, the authors of Tolonen et al imaged the entire MCE development, allowing for a complete and dynamic representation of the differentiation process. Using a combination of preexisting and custom-built segmentation and tracking technologies, they were able to create 3D masks of the epithelium, with some limitations stemming from variability in cell morphology and limited Z resolution. Importantly, nuclear tracking provided a reliable segmentation marker that allowed for accurate tracking of cell trajectories and was sufficient for creating single-cell trajectories that could be used for quantification of phenotypes within a lineage. The authors used this to define a morphodynamic state for each cell type through time to establish cell profiles using morphology and dynamics in a similar manner to how gene expression changes are used to define a molecular state (Copperman et al, 2023). Unfortunately, this initial analysis did not reveal distinct cell type clusters based on individual cellular features, showing no significant variances and highlighting the difficulties of analyzing compact and continuously changing tissues. This result is perhaps not too surprising, given that these are all epithelial cells and there are likely physical constraints to their morphology. Importantly however, these results fostered a shift in their analysis to supervised trained models where the authors used endpoint fixation and immunostaining of the tissue to establish a ground-truth dataset with assigned final cell identities that allowed them to backtrack cell lineages. When these supervised models were provided with lineage context, morphodynamic trends remained subtle but now became fate-associated. An expected outcome that validates their approach is that Z-position emerged as the strongest predictor due to the fact that certain cell types are only found at the apical surface of this multilayered tissue. However, other features such as the offset between nuclear and membrane centroids emerged as well, likely capturing changes in cell polarity and geometry during morphogenetic transitions such as radial intercalation.

While numerous groups have used supervised approaches, an important advance here was the use of multivariate multiclass verifiers with XGBoost and a multinomial logistic regression model from sci-kit to determine whether a combination of features could more accurately predict cell fate (Chen and Guestrin, 2016). In this case, XGBoost cell fate prediction had a mean accuracy of ~80% and when further tests were performed to determine which features contributed most significantly, Z-position again emerged as the greatest indicator. Though overall trends were subtle, with supervised models and multiple features being taken into account, quantitative morphodynamic analysis became an accurate predictor of cell fate (Fig. 1).

Single-cell molecular omics type measurements have helped inform our understanding of cell transitions and have, no doubt, ushered in an exciting time in developmental biology. However, to increase the resolution of our understanding of cell transitions, focusing on morphodynamics is a natural next step. Using a combination of live imaging and supervised models in a computational pipeline to predict cell fate will provide greater insights into this process and advance understanding of the functioning tissues and the cells they are composed of. It has recently been shown that single-cell phenomics provides insights into cell behavior during curved epithelia remodeling and the current work demonstrates that this data can be scaled and analyzed like other types of omics data, even in complex tissues (Stower et al, 2023).

In the future, coupling of morphodynamics with molecular approaches will allow more refined analysis of these transition points and promises to lead to significant advances. Additionally, single-cell phenotyping has been broadly used for diagnostics and development in biomedical research (De Vries et al, 2025), but being able to predict cell fate during differentiation will provide a baseline for developing new routes of early detection and intervention. Especially in fields like cancer research, understanding the morphodynamics of cell differentiation in full will be critical to catching divergences earlier than ever. Beyond the transformative current work from the Sedzinski lab, the field of morphodynamics is at a pivotal point where significant advances are occurring at a  remarkable pace.
